# Top 100 Cited Papers on Premenstrual Syndrome/Premenstrual Dysphoric Disorder: A Bibliometric Study

**DOI:** 10.3389/fpsyt.2022.936009

**Published:** 2022-07-14

**Authors:** Mingzhou Gao, Hao Zhang, Changlin Wang, Xiangyu Mou, Qingjun Zhu, Jieqiong Wang, Dongmei Gao

**Affiliations:** ^1^Team of Research and Innovation Focusing on Emotional Diseases and Syndromes, Innovation Research Institute of Traditional Chinese Medicine, Shandong University of Traditional Chinese Medicine, Jinan, China; ^2^Experimental Center, Shandong University of Traditional Chinese Medicine, Jinan, China; ^3^College of Traditional Chinese Medicine, Shandong University of Traditional Chinese Medicine, Jinan, China; ^4^School of Pharmacy, Shandong University of Traditional Chinese Medicine, Jinan, China

**Keywords:** bibliometric analysis, citations, premenstrual syndrome, trends, PMDD

## Abstract

**Background:**

Premenstrual syndrome/premenstrual dysphoric disorder is a serious condition affecting women worldwide, causing clinically significant distress or interference. Therefore, solving these diseases has become the utmost concern worldwide, culminating in numerous studies. In this study, we performed bibliometric analysis on the 100 most cited papers with the aim of identifying research hot spots and trends in this field.

**Methods:**

We screened the Science Citation Index Expanded (SCIE) of Web of Science (WOS) to identify the top 100 cited studies on PMS/PMDD. Next, we analyzed relevant literature from various journals, countries/regions, institutions, authors, and keywords. Finally, we used VOSviewer and Citespace software to generate knowledge maps and identify hot spots and trends.

**Results:**

The top 100 highly cited studies were published in 55 journals, between 1999 and 2017, across 24 countries/regions around the world. Most articles were published in Obstetrics and Gynecology, whereas Psych neuroendocrinology had the largest average number of citations per paper. The United States had the highest number of publications, followed by England, Canada, and Sweden. The top three institutions that published the highly cited literature were the University of Pennsylvania, Yale University, and National Institute of Mental Health (NIMH). Obstetrics, Gynecology, Psychiatry, and Reproductive Biology were the main research directions, whereas the top 10 Co-occurrence of Keywords included double-blind, fluoxetine, efficacy, prevalence, epidemiology, phase sertraline treatment, depression, progesterone, placebo, and placebo-controlled trial. Results from cluster analysis indicated that more comprehensive epidemiology and steroid pathogenesis have gradually become the hot spots and trends.

**Conclusion:**

These findings demonstrated that bibliometric analysis can intuitively and rapidly reveal the frontiers and hot spots of research in PMS/PMDD. Notably, epidemiology, steroid pathogenesis, GABAA receptor delta subunits, and double-blind placebo-controlled trials are potential areas of focus for future research.

## Introduction

Premenstrual dysphoric disorder (PMDD), a severe form of premenstrual syndrome (PMS), refers to a condition in which women of childbearing age exhibit periodic symptoms of discomfort, emotion, and physical disorders in the luteal phase, which deeply affect the patients' ability to study, and work as well as quality of life (1–5). Clinically, PMS/PMDD not only brings a substantial burden on both physical and mental aspects of life (6, 7) but also predisposes affected individuals to depression and bipolar disorders (8–10). Recent studies have demonstrated that some PMDD patients are suicidal, as evidenced by the fact that nearly 40% of PMDD women reported suicidal ideation (11, 12).

Published articles on PMS/PMDD date back to 1950 (13). In the past 60 years, researchers have made significant progress on PMS/PMDD, a phenomenon that is still ongoing (14). However, It is difficult to grasp the development overview and trend of PMS/PMDD research field, and scientific analysis of bibliometrics is urgently needed.

Knowledge on the top 100 cited papers may generate an understanding into the current focus of researchers. Bibliometric analysis, an effective method for analyzing literature, enhances researchers' understanding of a particular research area (15). In this study, we adopted bibliometric analysis to identify the top 100 cited papers on PMS/PMDD, and determined the trends in research on this topic from a perspective different from the previous research (14), which will promote the development of this research field and promote the basic research to gain new discoveries.

## Materials and Methods

### Data Sources and Search Strategies

Data were obtained from the Science Citation Index Expanded (SCI-E) database in the Web of Science Core Collection (WOSCC) (1). The literature search was framed as follows: [TI = (Premenstrual Syndrome) OR TI = (Premenstrual Dysphoric Disorder) OR TI = (late luteal phase dysphoric disorder) referring to related retrieval strategy (16). The range of article publication dates range was set from inception of the database to April 8th, 2022. No limitations were applied with regards to either the year of publication or language.

### Inclusion and Exclusion Criteria

Papers were displayed in descending order, based on the number of citations. Where necessary, two independent researchers identified the 100 most-cited papers after reading the titles, abstracts, and full texts. Conference papers and studies in which PMS/PMDD was not the central topic were excluded. Any disagreement, between the researchers, was resolved through discussion and consensus with a third researcher.

### Data Collection and Analysis

The top 100 articles were selected, then imported into Endnote X9, where several study characteristics, including title, keywords, document type, citation number, publication date, country, institutions, journals, and the 2020 impact factor of journals, were extracted. Next, we combined the online platform https://bibliometric.com, with VOSviewer 1.6.17, and Citespace R 5.8 to determine potential research hot spots and trends in this field by visualizing the aforementioned characteristics in the top 100 articles.

## Results

### Citation Characteristics of the Included Articles

**Figure 1:**
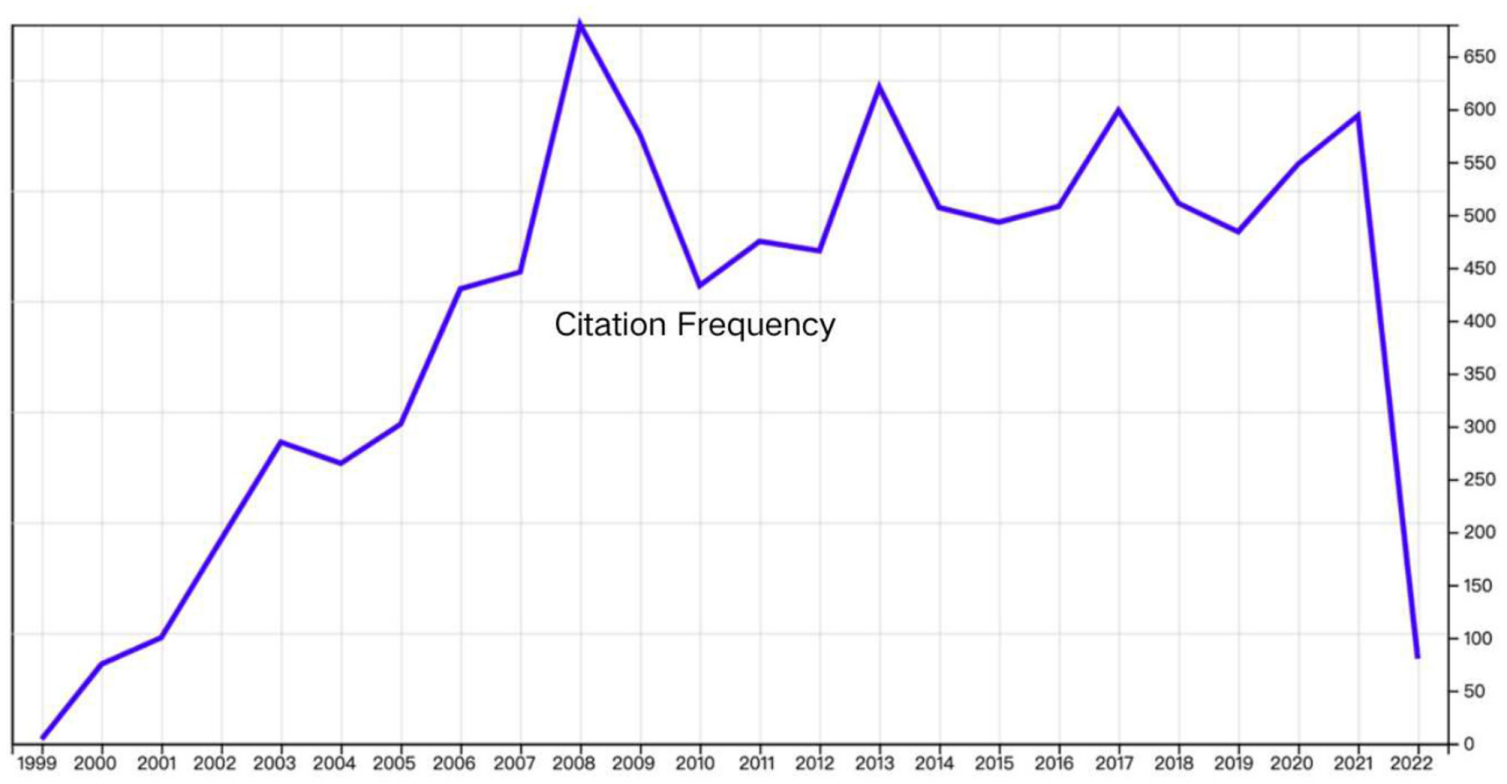
Frequency distribution of citations by year.

A total of 1,135 documents were initially retrieved from WoSCC. The list was arranged in descending order of citations, and the top 100 cited papers selected as listed in Table 1. The total number of citations for these 100 papers was 9,675 (median = 96.75) and frequency distribution of citations by year could been seen in Figure 1. The top 100 papers had 3821 references, with an h-index of 68. The most frequently cited articles included “The prevalence, impairment, impact, and burden of premenstrual dysphoric disorder (PMS/PMDD)” (17) by Halbreich, U (431 citations), followed by “Prevalence, incidence, and stability of premenstrual dysphoric disorder in the community” (355 citations) (18), “Cortical gamma-aminobutyric acid levels across the menstrual cycle in healthy women and those with premenstrual dysphoric disorder—A proton magnetic resonance spectroscopy study” (257 citations) (19), “Efficacy of a new low-dose oral contraceptive with drospirenone in premenstrual dysphoric disorder” (226 citations) (20) and “Efficacy of selective serotonin-reuptake inhibitors in premenstrual syndrome: a systematic review” (218 citations) (21). The abovementioned articles have been cited more than 200 times.

**Table 1:** The 100 most cited papers in PMS/PMDD until 2022.

**Rank**	**Title**	**First author**	**Journal**	**Year**	**Total citation**	**Average citation by year**
1	The prevalence, impairment, impact, and burden of premenstrual dysphoric disorder (PMS/PMDD)	Halbreich, U	PSYCHONEUROENDOCRINOLOGY	2003	431	21.55
2	Prevalence, incidence and stability of premenstrual dysphoric disorder in the community	Wittchen, HU	PSYCHOLOGICAL MEDICINE	2002	354	16.86
3	Premenstrual syndrome	Yonkers	LANCET	2008	257	17.13
4	Cortical gamma-aminobutyric acid levels across the menstrual cycle in healthy women and those with premenstrual dysphoric disorder—a proton magnetic resonance spectroscopy study	Epperson, CN	ARCHIVES OF GENERAL PSYCHIATRY	2002	257	12.24
5	Efficacy of a new low-dose oral contraceptive with drospirenone in premenstrual dysphoric disorder	Yonkers, KA	OBSTETRICS AND GYNECOLOGY	2005	226	12.56
6	Efficacy of selective serotonin-reuptake inhibitors in premenstrual syndrome: a systematic review	Dimmock, PW	LANCET	2000	218	9.48
7	Allopregnanolone levels and reactivity to mental stress in premenstrual dysphoric disorder	Girdler, SS	BIOLOGICAL PSYCHIATRY	2001	190	8.64
8	The etiology, biology, and evolving pathology of premenstrual syndromes	Halbreich, U	PSYCHONEUROENDOCRINOLOGY	2003	180	9
9	Treatment of premenstrual dysphoric disorder with a new drospirenone-containing oral contraceptive formulation	Pearlstein, TB	CONTRACEPTION	2005	177	9.83
10	Efficacy of vitamin B-6 in the treatment of premenstrual syndrome: systematic review	Wyatt, KM	BMJ-BRITISH MEDICAL JOURNAL	1999	167	6.96
11	Treatment for the premenstrual syndrome with agnus castus fruit extract: prospective, randomized, placebo controlled study	Schellenberg, R	BRITISH MEDICAL JOURNAL	2001	163	7.41
12	Premenstrual dysphoric disorder: evidence for a new category for DSM-5	Epperson, C	AMERICAN JOURNAL OF PSYCHIATRY	2012	161	14.64
13	Premenstrual syndrome and premenstrual dysphoric disorder: definitions and diagnosis	Freeman, EW	PSYCHONEUROENDOCRINOLOGY	2003	157	7.85
14	Allopregnanolone concentrations and premenstrual syndrome	Monteleone, P	EUROPEAN JOURNAL OF ENDOCRINOLOGY	2000	153	6.65
15	Biological, social, and behavioral factors associated with premenstrual syndrome	Deuster, PA	ARCHIVES OF FAMILY MEDICINE	1999	152	6.33
16	Premenstrual syndrome	Dickerson, LM	AMERICAN FAMILY PHYSICIAN	2003	143	7.15
17	Differential response to antidepressants in women with premenstrual syndrome/premenstrual dysphoric disorder–A randomized controlled trial	Freeman, EW	ARCHIVES OF GENERAL PSYCHIATRY	1999	130	5.42
18	A review of treatment of premenstrual syndrome & premenstrual dysphoric disorder	Rapkin, A	PSYCHONEUROENDOCRINOLOGY	2003	129	6.45
19	Prevalence and predictors of premenstrual dysphoric disorder (PMDD) in older premenopausal women—the harvard study of moods and cycles	Cohen, LS	JOURNAL OF AFFECTIVE DISORDERS	2002	126	6
20	Is premenstrual dysphoric disorder a distinct clinical entity?	Endicott, J	JOURNAL OF WOMENS HEALTH & GENDER-BASED MEDICINE	1999	119	4.96
21	Differential menstrual cycle regulation of hypothalamic-pituitary-adrenal axis in women with premenstrual syndrome and controls	Roca, CA	JOURNAL OF CLINICAL ENDOCRINOLOGY & METABOLISM	2003	118	5.9
22	Evaluation of a unique oral contraceptive in the treatment of premenstrual dysphoric disorder	Freeman, EW	JOURNAL OF WOMENS HEALTH & GENDER-BASED MEDICINE	2001	118	5.36
23	Clinical diagnostic criteria for premenstrual syndrome and guidelines for their quantification for research studies	Halbreich, Uriel	GYNECOLOGICAL ENDOCRINOLOGY	2007	116	7.25
24	The role of hormones and hormonal treatments in premenstrual syndrome	Backstrom, T	CNS DRUGS	2003	112	5.6
25	Calcium and vitamin D intake and risk of incident premenstrual syndrome	Bertone-Johnson, ER	ARCHIVES OF INTERNAL MEDICINE	2005	106	5.89
26	Changes in mood, cognitive performance and appetite in the late luteal and follicular phases of the menstrual cycle in women with and without PMDD (premenstrual dysphoric disorder)	Reed, Stephanie Collins	HORMONES AND BEHAVIOR	2008	104	6.93
27	Premenstrual daily fluoxetine for premenstrual dysphoric disorder: a placebo-controlled, clinical trial using computerized diaries	Cohen, LS	OBSTETRICS AND GYNECOLOGY	2002	104	4.95
28	Crocus sativus L. (saffron) in the treatment of premenstrual syndrome: a double-blind, randomized and placebo-controlled trial	Agha-Hosseini, M	BJOG-AN INTERNATIONAL JOURNAL OF OBSTETRICS AND GYNAECOLOGY	2008	102	6.8
29	Psychosocial functioning in women with premenstrual dysphoric disorder before and after treatment with sertraline or placebo	Pearlstein, TB	JOURNAL OF CLINICAL PSYCHIATRY	2000	101	4.39
30	Risk factors for premenstrual dysphoric disorder in a community sample of young women: the role of traumatic events and posttraumatic stress disorder	Perkonigg, A	JOURNAL OF CLINICAL PSYCHIATRY	2004	100	5.26
31	Selective serotonin reuptake inhibitors for premenstrual syndrome	Marjoribanks, Jane	COCHRANE DATABASE OF SYSTEMATIC REVIEWS	2013	99	9.9
32	Health and economic impact of the premenstrual syndrome	Borenstein, JE	JOURNAL OF REPRODUCTIVE MEDICINE	2003	99	4.95
33	Premenstrual syndrome, premenstrual dysphoric disorder, and beyond: a clinical primer for practitioners	Johnson, SR	OBSTETRICS AND GYNECOLOGY	2004	97	5.11
34	Oral contraceptives containing drospirenone for premenstrual syndrome	Lopez, Laureen M	COCHRANE DATABASE OF SYSTEMATIC REVIEWS	2012	96	8.73
35	Risk for premenstrual dysphoric disorder is associated with genetic variation in ESR1, the estrogen receptor alpha gene	Huo, Liang	BIOLOGICAL PSYCHIATRY	2007	93	5.81
36	Physiological changes during carbon dioxide inhalation in patients with panic disorder, major depression, and premenstrual dysphoric disorder—evidence for a central fear mechanism	Gorman, JM	ARCHIVES OF GENERAL PSYCHIATRY	2001	92	4.18
37	Toward a functional neuroanatomy of premenstrual dysphoric disorder	Protopopescu, Xenia	JOURNAL OF AFFECTIVE DISORDERS	2008	90	6
38	Premenstrual syndrome and premenstrual dysphoric disorder	Braverman, Paula K.	JOURNAL OF PEDIATRIC AND ADOLESCENT GYNECOLOGY	2007	90	5.63
39	The effectiveness of GnRHa with and without 'add-back' therapy in treating premenstrual syndrome: a meta analysis	Wyatt, KM	BJOG-AN INTERNATIONAL JOURNAL OF OBSTETRICS AND GYNAECOLOGY	2004	90	4.74
40	Premenstrual dysphoric disorder: burden of illness and treatment update	Pearlstein, Teri	JOURNAL OF PSYCHIATRY & NEUROSCIENCE	2008	89	5.93
41	Efficacy of intermittent, luteal phase sertraline treatment of premenstrual dysphoric disorder	Halbreich, U	OBSTETRICS AND GYNECOLOGY	2002	89	4.24
42	Gonadal steroid regulation of mood: the lessons of premenstrual syndrome	Rubinow, David R.	FRONTIERS IN NEUROENDOCRINOLOGY	2006	87	5.12
43	Prevalence of premenstrual syndrome and premenstrual dysphoric disorder in Japanese women	Takeda, T.	ARCHIVES OF WOMENS MENTAL HEALTH	2006	87	5.12
44	Efficacy of progesterone and progestogens in management of premenstrual syndrome: systematic review	Wyatt, K	BRITISH MEDICAL JOURNAL	2001	81	3.68
45	Pretreatment pattern of symptom expression in premenstrual dysphoric disorder	Pearlstein, T	JOURNAL OF AFFECTIVE DISORDERS	2005	80	4.44
46	How does premenstrual dysphoric disorder relate to depression and anxiety disorders?	Landen, M	DEPRESSION AND ANXIETY	2003	80	4
47	Prevalence of menstrual cycle symptom cyclicity and premenstrual dysphoric disorder in a random sample of women using and not using oral contraceptives	Sveindottir, H	ACTA OBSTETRICIA ET GYNECOLOGICA SCANDINAVICA	2000	80	3.48
48	Luteal phase sertraline treatment for premenstrual dysphoric disorder—results of a double-blind, placebo-controlled, crossover study	Jermain, DM	ARCHIVES OF FAMILY MEDICINE	1999	80	3.33
49	Premenstrual dysphoric disorder: epidemiology and treatment	Hantsoo, Liisa	CURRENT PSYCHIATRY REPORTS	2015	79	9.88
50	Steroid withdrawal in the mouse results in anxiogenic effects of 3 alpha, 5 beta-THP: a possible model of premenstrual dysphoric disorder	Smith, Sheryl S	PSYCHOPHARMACOLOGY	2006	79	4.65
51	Premenstrual syndrome as a predictor of menopausal symptoms	Freeman, EW	OBSTETRICS AND GYNECOLOGY	2004	79	4.16
52	The diagnosis of premenstrual syndromes and premenstrual dysphoric disorder—clinical procedures and research perspectives	Halbreich, U	GYNECOLOGICAL ENDOCRINOLOGY	2004	77	4.05
53	Premenstrual syndrome and premenstrual dysphoric disorder	Biggs, Wendy S	AMERICAN FAMILY PHYSICIAN	2011	76	6.33
54	Sleep, hormones, and circadian rhythms throughout the menstrual cycle in healthy women and women with premenstrual dysphoric disorder	Shechter, Ari	INTERNATIONAL JOURNAL OF ENDOCRINOLOGY	2010	76	5.85
55	Premenstrual syndrome and premenstrual dysphoric disorder: guidelines for management	Steiner, M	JOURNAL OF PSYCHIATRY & NEUROSCIENCE	2000	76	3.3
56	Treatment of premenstrual syndrome with gonadotropin-releasing hormone agonist in a low dose regimen	Sundstrom, I	ACTA OBSTETRICIA ET GYNECOLOGICA SCANDINAVICA	1999	76	3.17
57	Premenstrual syndrome: a mini review	Ryu, Aeli	MATURITAS	2015	75	9.38
58	Premenstrual syndrome and associated symptoms in adolescent girls	Derman, O	EUROPEAN JOURNAL OF OBSTETRICS & GYNECOLOGY AND REPRODUCTIVE BIOLOGY	2004	75	3.95
59	Fluoxetine vs. Vitex agnus castus extract in the treatment of premenstrual dysphoric disorder	Atmaca, M	HUMAN PSYCHOPHARMACOLOGY-CLINICAL AND EXPERIMENTAL	2003	75	3.75
60	Prevalence of sexual abuse history in a sample of women seeking treatment for premenstrual syndrome	Golding, JM	JOURNAL OF PSYCHOSOMATIC OBSTETRICS AND GYNECOLOGY	2000	75	3.26
61	Selective serotonin reuptake inhibitors for premenstrual syndrome and premenstrual dysphoric disorder—a meta-analyslis	Shah, Nirav R	OBSTETRICS AND GYNECOLOGY	2008	74	4.93
62	Venlafaxine in the treatment of premenstrual dysphoric disorder	Freeman, EW	OBSTETRICS AND GYNECOLOGY	2001	74	3.36
63	Menstrual cycle effects on amygdala reactivity to emotional stimulation in premenstrual dysphoric disorder	Gingnell, Malin	HORMONES AND BEHAVIOR	2012	73	6.64
64	Randomized controlled trial of the management of premenstrual syndrome and premenstrual mastalgia using luteal phase-only danazol	O'Brien, PMS	AMERICAN JOURNAL OF OBSTETRICS AND GYNECOLOGY	1999	73	3.04
65	Prevalence and predictors of premenstrual syndrome and premenstrual dysphoric disorder in a population-based sample	Tschudin, Sibil	ARCHIVES OF WOMENS MENTAL HEALTH	2010	72	5.54
66	Sleep quality and the sleep electroencephalogram in women with severe premenstrual syndrome	Baker, Fiona C	SLEEP	2007	70	4.38
67	Estimating direct and indirect costs of premenstrual syndrome	Borenstein, J	JOURNAL OF OCCUPATIONAL AND ENVIRONMENTAL MEDICINE	2005	70	3.89
68	Diagnosis and treatment of premenstrual dysphoric disorder: an update	Steiner, M	INTERNATIONAL CLINICAL PSYCHOPHARMACOLOGY	2000	70	3.04
69	Weekly luteal-phase dosing with enteric-coated fluoxetine 90 mg in premenstrual dysphoric disorder: a randomized, double-blind, placebo-controlled clinical trial	Miner, C	CLINICAL THERAPEUTICS	2002	68	3.24
70	Micronutrients and the premenstrual syndrome: the case for calcium	Thys-Jacobs, S	JOURNAL OF THE AMERICAN COLLEGE OF NUTRITION	2000	66	2.87
71	The prevalence of premenstrual dysphoric disorder in a randomly selected group of urban and rural women	Gehlert, S	PSYCHOLOGICAL MEDICINE	2009	65	4.64
72	Allopregnanolone levels and symptom improvement in severe premenstrual syndrome	Freeman, EW	JOURNAL OF CLINICAL PSYCHOPHARMACOLOGY	2002	64	3.05
73	Abnormal luteal phase excitability of the motor cortex in women with premenstrual syndrome	Smith, MJ	BIOLOGICAL PSYCHIATRY	2003	62	3.1
74	Premenstrual dysphoric disorder	Grady-Weliky, TA	NEW ENGLAND JOURNAL OF MEDICINE	2003	62	3.1
75	Premenstrual dysphoric disorder symptoms following ovarian suppression: triggered by change in ovarian steroid levels but not continuous stable levels	Schmidt, Peter J	AMERICAN JOURNAL OF PSYCHIATRY	2017	61	10.17
76	Association of inflammation markers with menstrual symptom severity and premenstrual syndrome in young women	Bertone-Johnson, E. R	HUMAN REPRODUCTION	2014	61	6.78
77	Obesity as a risk factor for premenstrual syndrome	Masho, SW	JOURNAL OF PSYCHOSOMATIC OBSTETRICS & GYNECOLOGY	2005	61	3.39
78	Continuous or intermittent dosing with sertraline for patients with severe premenstrual syndrome or premenstrual dysphoric disorder	Freeman, EW	AMERICAN JOURNAL OF PSYCHIATRY	2004	61	3.21
79	A randomized comparison of psychological (cognitive behavior therapy), medical (fluoxetine) and combined treatment for women with premenstrual dysphoric disorder	Hunter, MS	JOURNAL OF PSYCHOSOMATIC OBSTETRICS & GYNECOLOGY	2002	61	2.9
80	Premenstrual dysphoric disorder—is there an economic burden of illness?	Chawla, A	MEDICAL CARE	2002	60	2.86
81	Abnormalities of dorsolateral prefrontal function in women with premenstrual dysphoric disorder: a multimodal neuroimaging study	Baller, Erica B	AMERICAN JOURNAL OF PSYCHIATRY	2013	59	5.9
82	Biological correlates of abuse in women with premenstrual dysphoric disorder and healthy controls	Girdler, SS	PSYCHOSOMATIC MEDICINE	2003	59	2.95
83	Premenstrual syndrome prevalence and fluctuation over time: results from a french population-based survey	Potter, Julia	JOURNAL OF WOMENS HEALTH	2009	58	4.14
84	A controlled study of light therapy in women with late luteal phase dysphoric disorder	Lam, RW	PSYCHIATRY RESEARCH	1999	56	2.42
85	Brain-derived neurotrophic factor plasma variation during the different phases of the menstrual cycle in women with premenstrual syndrome	Cubeddu, Alessandra	PSYCHONEUROENDOCRINOLOGY	2011	56	4.67
86	Placebo-controlled trial comparing intermittent and continuous paroxetine in premenstrual dysphoric disorder	Landen, Mikael	NEUROPSYCHOPHARMACOLOGY	2007	56	3.5
87	Are there differential symptom profiles that improve in response to different pharmacological treatments of premenstrual syndrome/premenstrual dysphoric disorder?	Halbreich, Uriel	CNS DRUGS	2006	56	3.29
88	Specificity of panic response to CO2 inhalation in panic disorder: a comparison with major depression and premenstrual dysphoric disorder	Kent, JM	AMERICAN JOURNAL OF PSYCHIATRY	2001	56	2.55
89	Characteristics of placebo responses in medical treatment of premenstrual syndrome	Freeman, EW	AMERICAN JOURNAL OF PSYCHIATRY	1999	56	2.33
90	Premenstrual syndrome and premenstrual dysphoric disorder	Hofmeister, Sabrina	AMERICAN FAMILY PHYSICIAN	2016	55	7.86
91	Cognitive-behavioral therapy for premenstrual syndrome and premenstrual dysphoric disorder: a systematic review	Lustyk, M. Kathleen B	ARCHIVES OF WOMENS MENTAL HEALTH	2009	54	3.86
92	Luteal-phase accentuation of acoustic startle response in women with premenstrual dysphoric disorder	Epperson, Cynthia Neill	NEUROPSYCHOPHARMACOLOGY	2007	54	3.38
93	Hysterectomy and bilateral oophorectomy for severe premenstrual syndrome	Cronje, WH	HUMAN REPRODUCTION	2004	54	2.84
94	5 alpha-reductase inhibition prevents the luteal phase increase in plasma allopregnanolone levels and mitigates symptoms in women with premenstrual dysphoric disorder	Martinez, Pedro E	NEUROPSYCHOPHARMACOLOGY	2016	53	7.57
95	Premenstrual syndrome—advances in diagnosis and treatment	Kessel, B	OBSTETRICS AND GYNECOLOGY CLINICS OF NORTH AMERICA	2000	53	2.3
96	Proton magnetic resonance spectroscopy measurement of brain glutamate levels in premenstrual dysphoric disorder	Batra, Neha Arun	BIOLOGICAL PSYCHIATRY	2008	52	3.47
97	The treatment of severe premenstrual syndrome with goserelin with and without 'add-back' estrogen therapy: a placebo-controlled study	Leather, AT	GYNECOLOGICAL ENDOCRINOLOGY	1999	51	2.13
98	Prevalence, impacts and medical managements of premenstrual syndrome among female students: cross-sectional study in college of health sciences, mekelle university, mekelle, northern ethiopia	Tolossa, Fikru Wakjira	BMC WOMENS HEALTH	2014	50	5.56
99	Full- or half-cycle treatment of severe premenstrual syndrome with a serotonergic antidepressant	Freeman, EW	JOURNAL OF CLINICAL PSYCHOPHARMACOLOGY	1999	50	2.08
100	Factors associated with premenstrual syndrome-A survey of new female university students	Cheng, Shu-Hu	KAOHSIUNG JOURNAL OF MEDICAL SCIENCES	2013	49	4.9

### Year and Types of Publication

**Figure 2:**
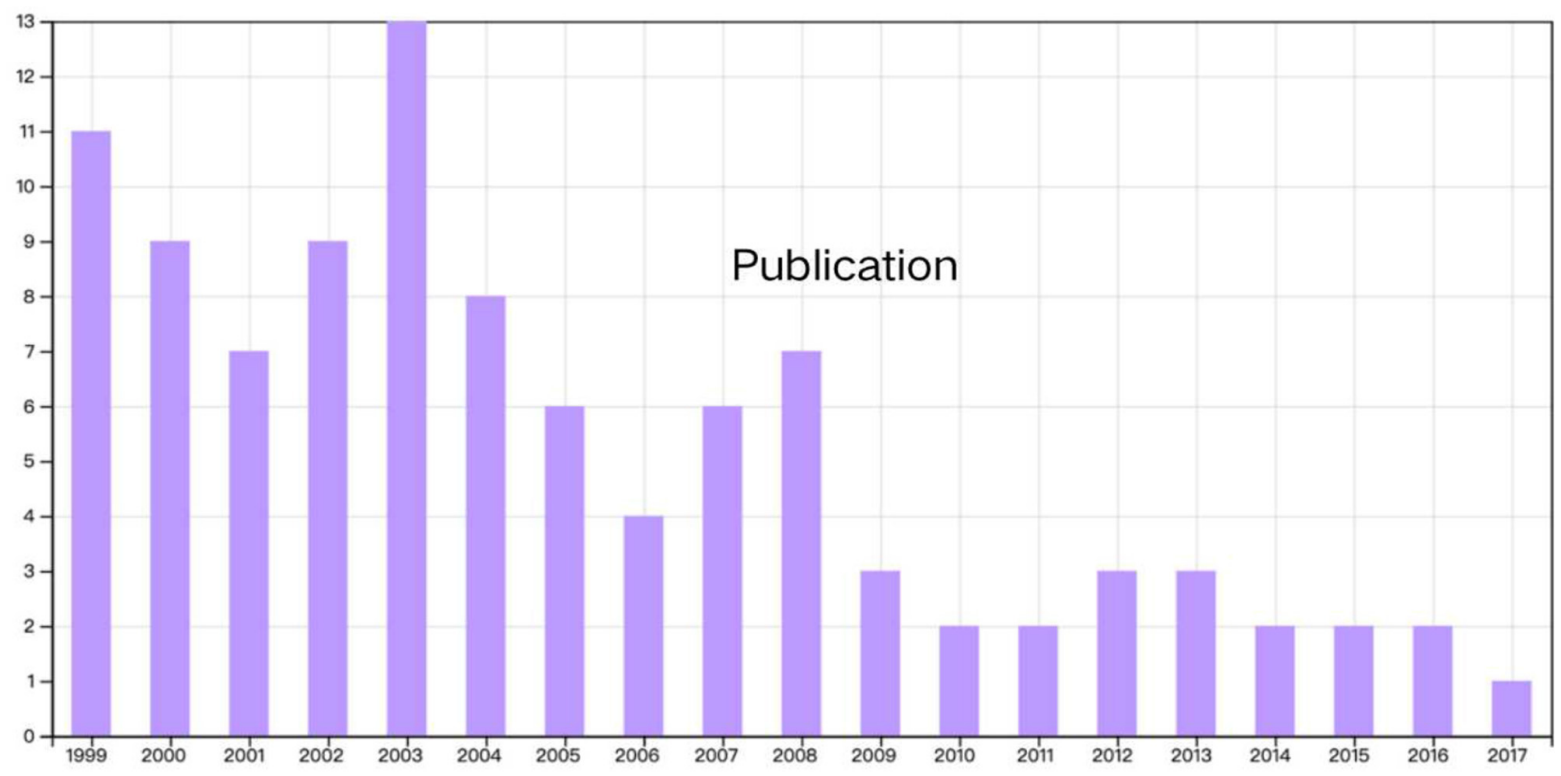
Distribution of articles by year of publication.

The distribution of publications by year is illustrated using a histogram in Figure 2. The top 100 cited papers on PMS/PMDD were published between 1999 and 2017, although articles published between 2018 and 2022 were not included in the list. Additionally, 13 papers published in 2007 had the most significant impact on research in this field. Among the selected papers, 67 and 33 were original research articles and reviews, respectively. Furthermore, the 23 papers published in 2003 had the most significant impact on research in this field (Figure 2).

### Distribution Per Journal

**Figure 3:**
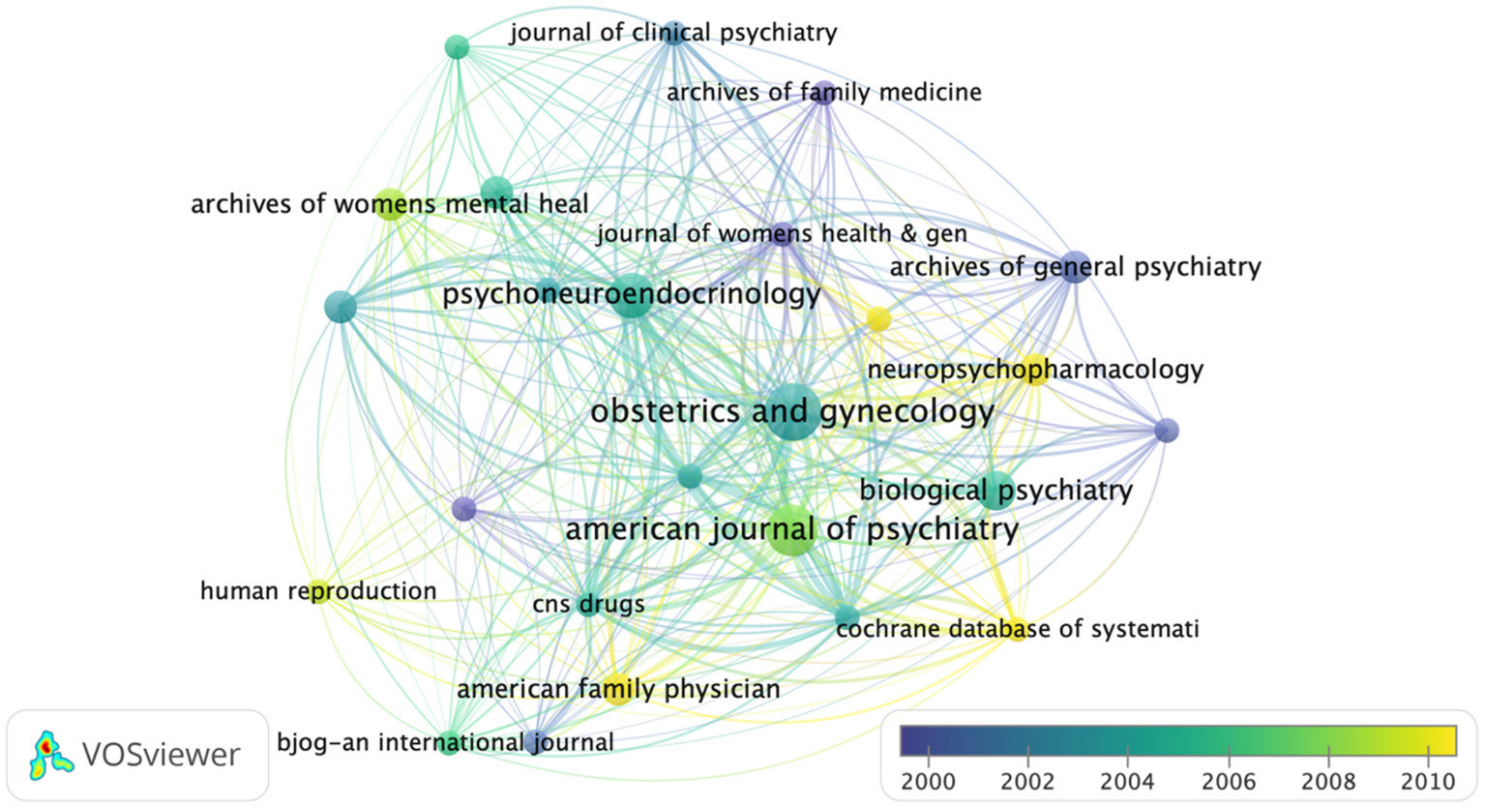
Network-based visualization of journals that published the 100 most cited articles according to average publication year. The size of the circles represents the number of articles in the 100 most cited list, whereas the width of the curved line denotes link strength. The distance between 2 journals indicates the approximate relatedness of the nodes.

We generated a citation network of journals, based on the average published year, to depict interaction among the top 100 cited articles published in 55 journals (Figure 3). Notably, Obstetrics and Gynecology published the largest number of papers (7 papers), followed by the American journal of psychiatry (6 papers). On the other hand, Psych neuroendocrinology had the largest average number of citations per paper (1 paper, 917 citations).

### Contributing Authors

**Figure 4:**
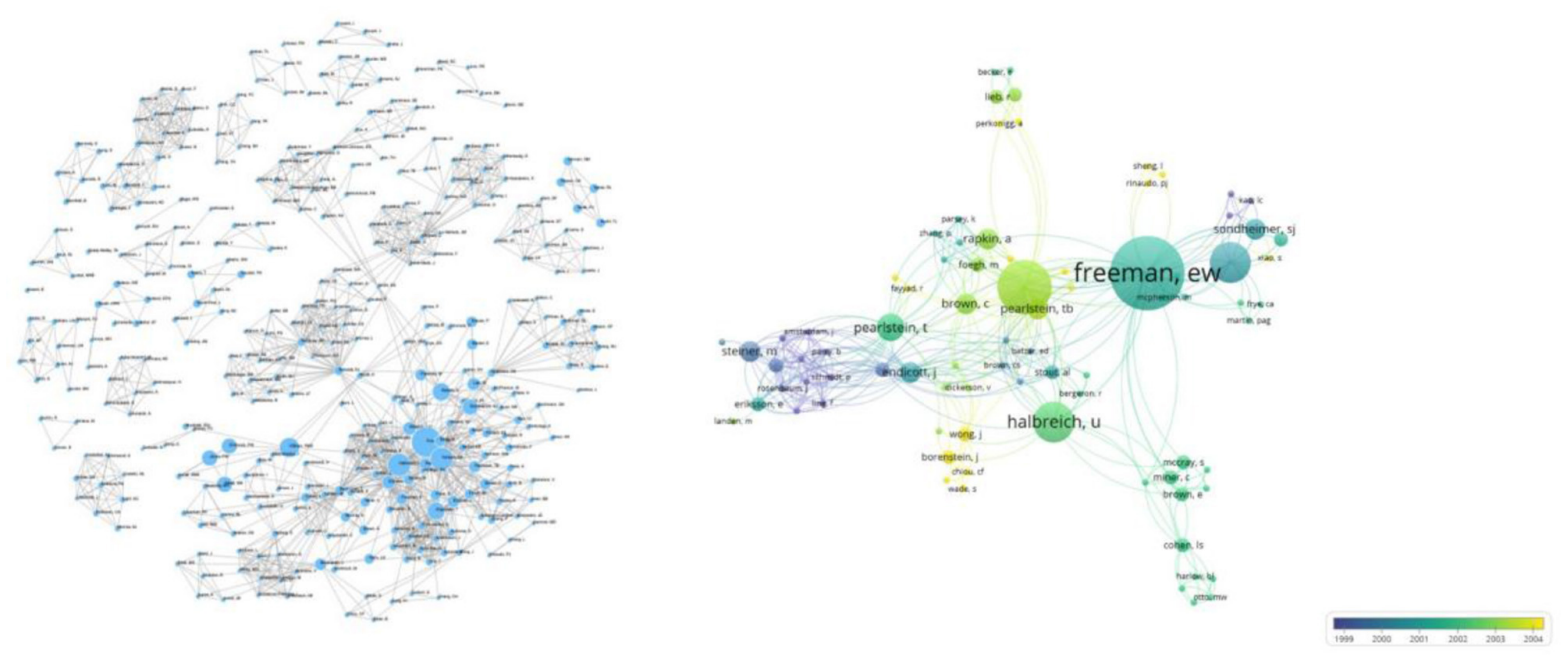
Networks showing interconnectivity among co-authors across the 100 most cited articles according to the average published year. Size of the circles indicate the number of articles in the 100 most cited list, while the width of the curved line represents the link strength. The distance between two authors indicates approximate relatedness among the nodes.

A total of 360 authors contributed to the top 100 cited papers, although some of the 386 items in the network were not connected. The largest set of connected items comprised 83 items. Notably, Freeman EW was at the core of this network, although Halbreich U and Yonkers KA emerged as new authors in recent years. Freeman EW had the highest number of papers (11, with 956 citations), followed by Halbreich U and Yonkers KA who had 645 and 894 citations, respectively (Figure 4).

### Contributing Countries/Regions and Institutions

**Figure 5:**
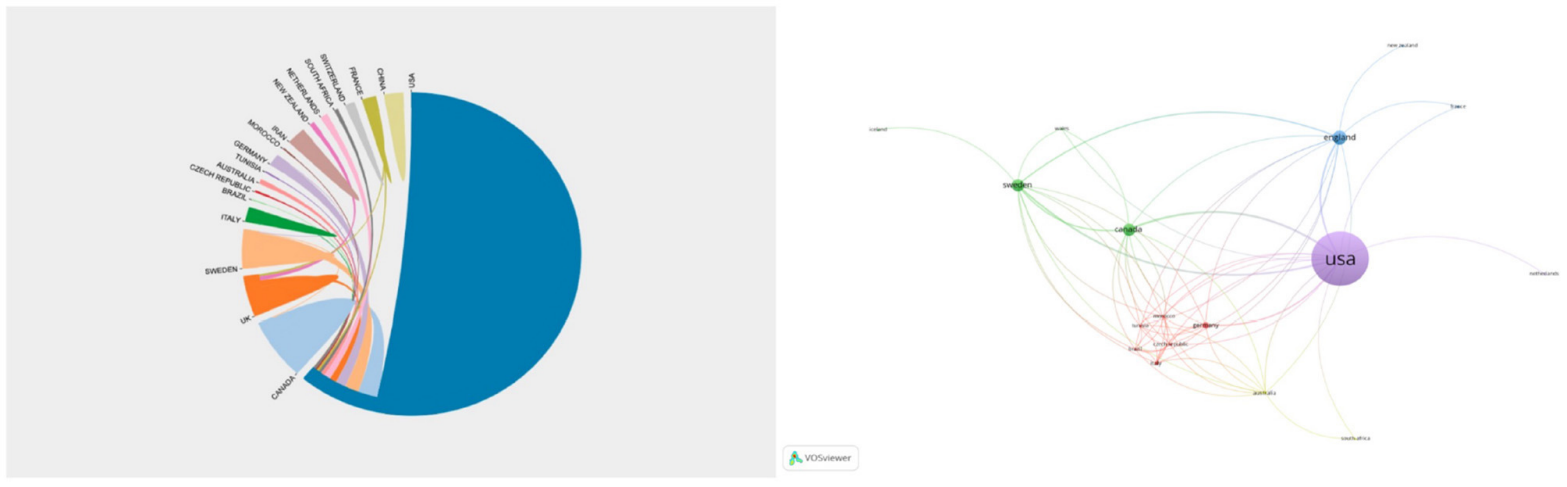
Networks showing the collaboration among countries/regions in the top 100 cited papers. Circle size represents the number of papers in the top 100 cited articles.

**Figure 6:**
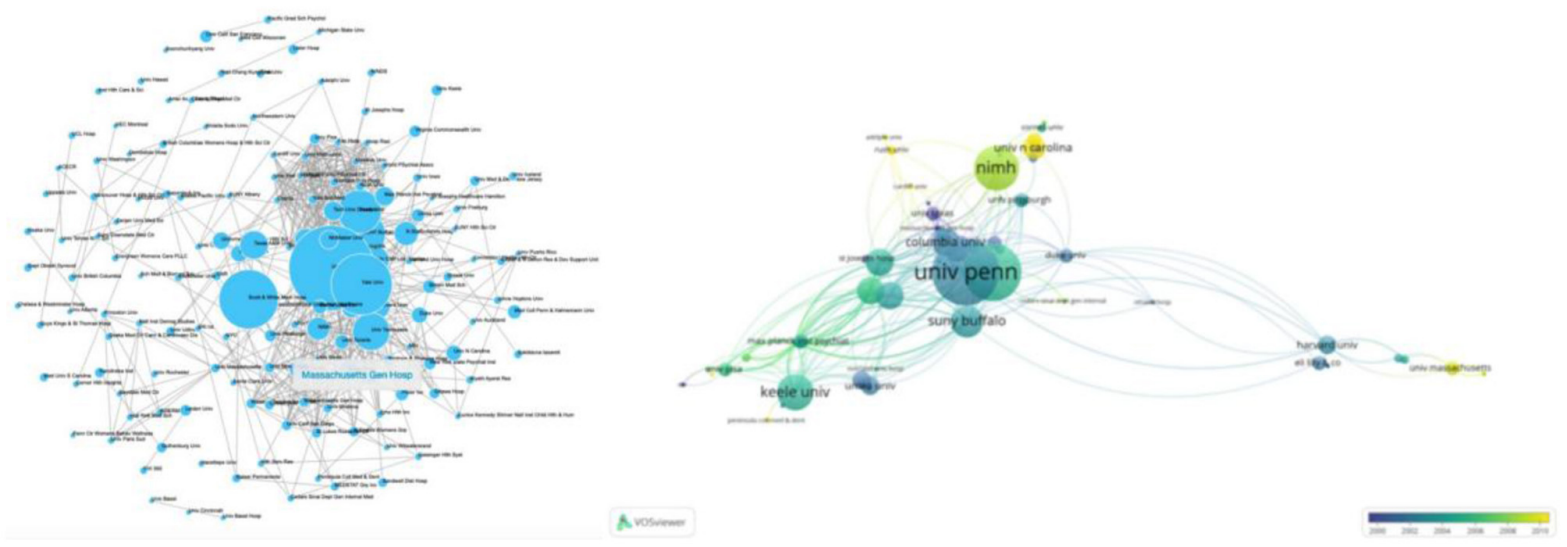
Network visualization of the institutions that contributed to the top 100 cited articles. The size of the circle represents the number of papers in the top 100 list.

The selected top 100 cited papers were published across 24 countries or regions (Figure 5). Among them, the United States had the highest contribution (68 articles), followed by England (13 papers). Among the 24 countries, the United States formed the largest national cooperation network, covering 17 countries. In terms of research institutions, the 100 most cited papers were published by 153 institutions, with the largest set of connected items comprising 107 institutions. Specifically, the University of Pennsylvania contributed the most papers (15 papers), followed by Yale University and NiMH which accounted for 13 and 10 papers, respectively (Figure 6).

### Research Direction

The top 100 cited papers on PMS/PMDD were stratified into various study directions based on WOS categories as shown in Table 2. Among them, “Obstetrics Gynecology” (98 papers) was the topmost research direction, followed by “Psychiatry” (63 papers), and “Reproductive Biology” (61 papers), among others (Table 2).

**Table 2:** Top 10 research directions identified in the 100 most cited papers on PMS/PMDD.

**Rank**	**Research direction**	**No. of papers**
1	Obstetrics gynecology	98
2	Psychiatry	63
3	Reproductive biology	61
4	Behavioral sciences	60
5	Pharmacology pharmacy	60
6	Psychology	49
7	Neurosciences neurology	46
8	Endocrinology metabolism	40
9	Health care sciences services	29
10	Biochemistry molecular biology	26

### Co-occurrence of Keywords

**Figure 7:**
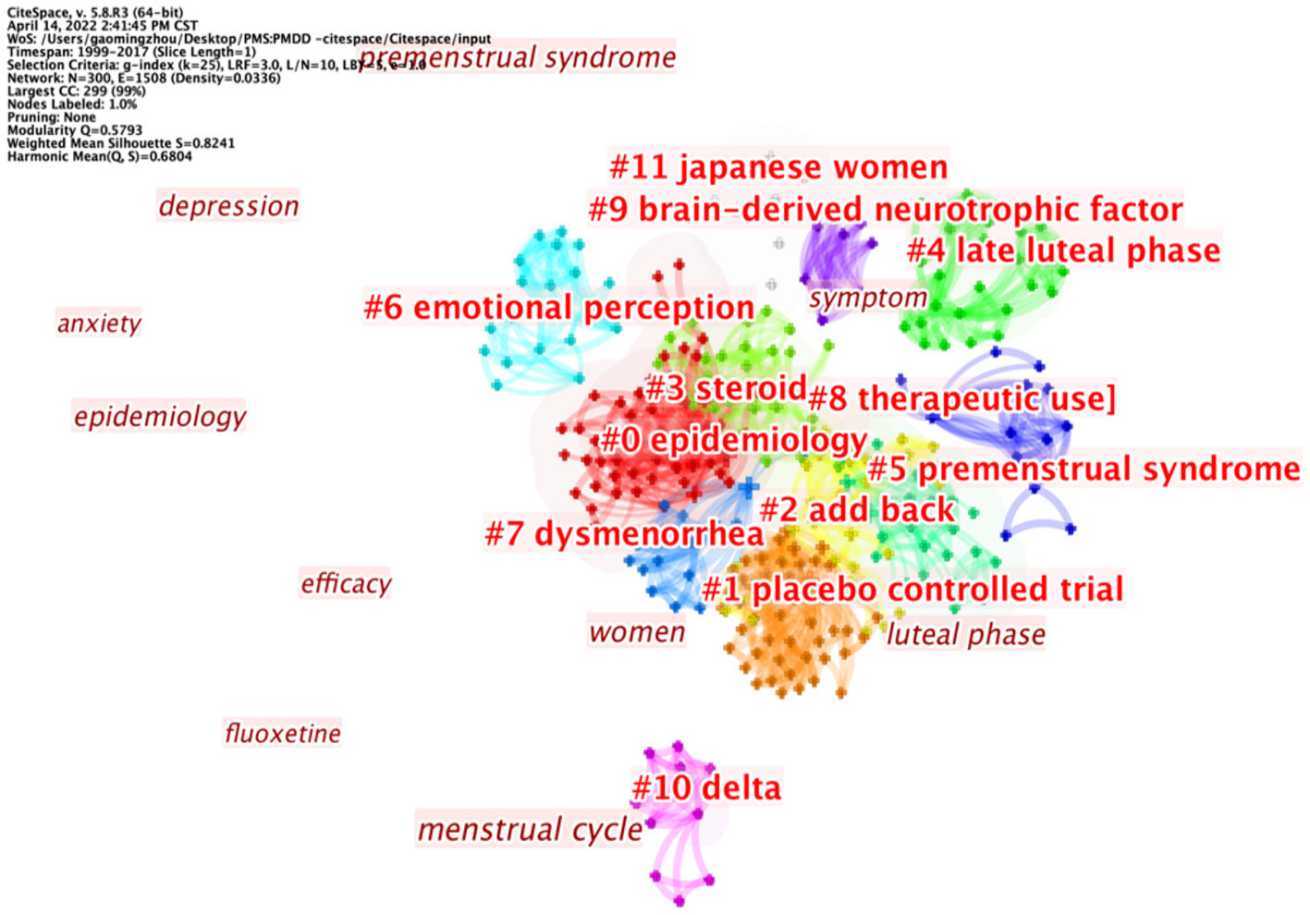
Network showing interaction among cooccurring keywords in the top 100 cited papers.

A total of 477 keywords were identified across the top 100 cited papers. We excluded 10 keywords, such as women, menstrual cycle, premenstrual dysphoric disorder, and premenstrual syndrome, among others. Further analysis revealed cooccurring keywords, with the following forming the top 10; double-blind, fluoxetine, efficacy, prevalence, epidemiology, phase sertraline treatment, depression, progesterone, placebo, and placebo-controlled trial (Table 3). These keywords were further classified into 11 clusters as follows: cluster 1 (epidemiology), cluster 2 (placebo-controlled trial), cluster 3 (steroid), cluster 4 (late luteal phase), cluster 5(premenstrual syndrome), cluster 6 (emotional perception), cluster 7 (dysmenorrhea), cluster 8 (therapeutic use), cluster 9 (brain-derived neurotrophic factor), cluster 10 (delta) and cluster 11 (Japanese women) (Figure 7, Table 4).

**Table 3:** Top 10 Co-occurring Keywords in the Top 100 cited papers on PMS/PMDD.

**Rank**	**Keyword**	**Occurrences**
1	Double-blind	29
2	Fluoxetine	15
3	Efficacy	14
4	Prevalence	14
5	Epidemiology	13
6	Phase sertraline treatment	13
7	Depression	12
8	Progesterone	12
9	Placebo	11
10	Placebo-controlled trial	11

**Table 4:** Clusters of keywords cooccurring in the top 100 cited papers on PMS/PMDD.

**Cluster-ID**	**Size**	**Mean (Year)**	**Label (LLR)**
0	56	2004	Epidemiology (11.78, 0.001); prevalence (7.41, 0.01); menstruation (6.66, 0.01); productivity (6.66, 0.01); depression (5.93, 0.05)
1	50	2004	Placebo controlled trial (17.74, 1.0E-4); crossover (10.59, 0.005); luteal phase (10.09, 0.005); randomized controlled trial (9.52, 0.005); fluoxetine treatment (7.04, 0.01)
2	31	2005	Add back (12.08, 0.001); depot leuprolide (8.03, 0.005); double blind crossover (8.03, 0.005); management (8.03, 0.005); estrogen (8.03, 0.005)
3	30	2004	Steroid (10.79, 0.005); glutamate (10.04, 0.005); progesterone (7.29, 0.01); allopregnanolone (6.39, 0.05); 5 alpha pregnane 3,20 dione (5, 0.05)
4	28	2006	Late luteal phase (6.31, 0.05); estrogen receptor alpha gene (6.31, 0.05); gonadal steroids (6.31, 0.05); esr1 (6.31, 0.05); core temperature (6.31, 0.05)
5	24	2000	Premenstrual syndrome (6.77, 0.01); luteal administration (4.15, 0.05); phototherapy (4.15, 0.05); seasonal affective disorder (4.15, 0.05); research (4.15, 0.05)
6	19	2006	Emotional perception (7.49, 0.01); challenge test (7.49, 0.01); attack (7.49, 0.01); lactate infusion (7.49, 0.01); fmri (7.49, 0.01)
7	17	2011	Dysmenorrhea (4.12, 0.05); adolescence (4.12, 0.05); combined (4.12, 0.05); modulator (4.12, 0.05); vitex agnus castus (4.12, 0.05)
8	16	2008	Therapeutic use (11.9, 0.001); cycle control (5.91, 0.05); randomized controlled trials as topic (5.91, 0.05); contraceptives oral combined [adverse effects (5.91, 0.05); sleep spindles (5.91, 0.05)
9	10	2009	Brain-derived neurotrophic factor (10.04, 0.005); sex hormones (10.04, 0.005); menstrual cycle (2, 0.5); premenstrual syndrome (1.93, 0.5); women (0.31, 1.0)
10	10	2006	Delta (7.84, 0.01); alpha-4 (7.84, 0.01); mouse (7.84, 0.01); thip (7.84, 0.01); elevated plus maze (7.84, 0.01)
11	8	2000	Japanese women (8.8, 0.005); womens health (8.8, 0.005); sexual abuse (8.8, 0.005); post-traumatic stress disorder (8.8, 0.005); pms (2.71, 0.1)

## Discussion

Women's menstrual cycle-related disorders, such as premenstrual syndrome and dysmenorrhea, predispose many women to various diseases which subsequently adversely affect their health (22).

### General Information From the Top 100 Most-Cited Papers on Top 100 Most-Cited Papers

The top 100 cited papers were selected from papers titled “Premenstrual Syndrome,” “Premenstrual Dysphoric Disorder,” or “late luteal phase dysphoric disorder” referring to its citations, which represent the concerns of researchers. Analysis of the selected top 100 cited articles resulted in a total of 9,676 citations, with an average of 96.76 citations per article. Among them, the earliest paper entitled “Randomized controlled trial of the management of premenstrual syndrome and premenstrual mastalgia using luteal phase-only danazol,” was published in 1999 (23), whereas the most recent one was “Premenstrual Dysphoric Disorder Symptoms Following Ovarian Suppression: Triggered by Change in Ovarian Steroid Levels but Not Continuous Stable Levels” published in 2017 (24). Moreover, “The prevalence, impairment, impact, and burden of premenstrual dysphoric disorder (PMS/PMDD)” (17) by Halbreich was the most cited article.

### Influential Authors and Cooperative Network

Analysis of the 100 most cited papers revealed contribution by 386 authors. Among them, Freeman EW, Halbreich U, and Yonkers KA were the highest contributors to the most influential articles, suggesting that their researches are a potential hotspot in this field. Particularly, Freeman EW's s newest paper, entitled “Are there differential symptom profiles that improve in response to different pharmacological treatments of premenstrual syndrome/premenstrual dysphoric disorder?” revealed possible well-defined subgroups of PMDD. It is evident that overall treatment response rates may improve if treatments are targeted at subtypes (25). On the other hand, Yonkers KA's paper, entitled “Premenstrual Dysphoric Disorder: Evidence for a New Category for DSM-5,” discussed DSM-5 for premenstrual dysphoric disorder (26), whereas Halbreich U's article “Clinical diagnostic criteria for premenstrual syndrome and guidelines for their quantification for research studies,” revealed updated diagnostic criteria for PMS/PMDD and guidelines for clinical and research applications (27). A total of 83 items showed an interaction, of which Freeman EW was at the core of this network, although new scholars, such as Halbreich U and Yonkers KA have emerged in recent years. These authors are distributed across 24 countries or regions and 153 institutions. Among them, the University of Pennsylvania and the United States contributed the highest number of publications. This indicated that American institutions and authors play a leading role in research on PMDD, this may guide future research activities.

### Future Perspectives

#### Epidemiology for Prevalence and Risk Factors

Epidemiological research is the primary and critical approach for exploring development and progression of PMS/PMDD. Notably, 11 out of the 100 most cited articles described prevalence of PMS/PMDD between 2000 and 2014 (17, 18, 28–35), while 29 focused on epidemiology. Collectively, these articles provide reliable data sources to better our understanding of PMS/PMDD, including incidence rate. In recent years, numerous studies have evaluated childhood body size and premenstrual disorders in young adulthood (36), comorbid bipolar disorder (37), prevalence, and associated factors among different groups (38). For instance, studies showed that the prevalence of PMS among Academics at a University in Midwest Brazil was 46.9% (38), and 21.1% for university students (39), which are higher than ever before. Moreover, risk factors for PMS/PMDD include childhood abuse and neglect (40), childhood maltreatment (41), and perinatal depression (42), among others.

#### Steroid Pathogenesis

At present, the pathogenesis of PMDD remains unclear. However, steroid pathogenesis such as progesterone and allopregnanolone in PMS/PMDD has always been research hotspots. New findings have suggested that progesterone exerts a different effect on the metabolic profiles of women with PMDD compared to controls (43). In addition, a change in estradiol/progesterone levels from low to high, and not the steady-state level, was associated with onset of PMDD symptoms (24). Gradually, researchers have found that allopregnanolone is the provoking factor behind the negative mood symptoms in PMDD, a disease whose pathophysiology is significantly correlated with impaired GABAA-R response to dynamic ALLO fluctuations across the menstrual cycle, manifesting in affective symptoms (44–46). It is possible that GABAA-R response to allopregnanolone, alpha4 and delta subunits of GABAA-R may be playing an essential role in mood swings (47). Recent studies have also associated copy number variations in GABRB2 with PMDD (48, 49). Future studies are expected to elucidate GABAA-R's susceptibility to ALLO.

#### Placebo-Controlled Trials for Treatment of PMS/PMDD

Treatment is a crucial step in management of PMS/PMDD. The clusters of co-occurrent keywords suggest that prospecting for effective treatment therapies for PMS/PMDD, such as use of fluoxetine (50), and vitex agnus castus (51), is a promising research hotspot. However, placebo-controlled trials are needed to generate more reliable data.

## Limitations

This study had several limitations. After consulting numerous literature, we used the Web of Science to identify relevant datasets. Although this is the most commonly used database for literature searches, some early publications may be missing. For an accurate econometric analysis, we referred to previous studies (16) and adopted a Title keyword rather than Topic keyword retrieval approach. Although our search results were accurate, they may not be extensive enough.

## Conclusion

To the best of our knowledge, this is the first bibliometric analysis of the most frequently cited papers on PMS/PMDD. Our results indicate that most essential studies on PMS/PMDD have been published in the Journal of Obstetrics and Gynecology. American authors and institutions have played a leading role in research on PMDD, thus represent a future research direction. Moreover, epidemiology for prevalence and risk factors, steroid pathogenesis, such as progesterone, allopregnanolone, and placebo-controlled trial for the treatment represent future trends in this field of research.

## Author Contributions

MG designed the study and wrote and revised the draft manuscript. MG, HZ, CW, and XM performed literature search, retrieval, and data collection. MG and HZ carried out data visualization and graphical interpretation. QZ, DG, and JW provided critical assistance or funding. All authors contributed to and approved the final draft of the manuscript before submission.

## Funding

This study was supported by funds from the Key Project of Natural Science Foundation of Shandong Province (No. ZR2020ZD17), National Natural Science Foundation of China (Nos. 81001484 and 81473558), Natural Science Foundation of Shandong Province (No. ZR202102270167), Shandong medical and health science and technology development plan project (No. 202105010467) and 20 articles for colleges and universities funded project in Jinan (No. 2020GXRC002).

## Conflict of Interest

The authors declare that the research was conducted in the absence of any commercial or financial relationships that could be construed as a potential conflict of interest.

## Publisher's Note

All claims expressed in this article are solely those of the authors and do not necessarily represent those of their affiliated organizations, or those of the publisher, the editors and the reviewers. Any product that may be evaluated in this article, or claim that may be made by its manufacturer, is not guaranteed or endorsed by the publisher.
